# Ex Vivo Biomechanical Bone Testing of Pig Femur as an Experimental Model

**DOI:** 10.3390/bioengineering11060572

**Published:** 2024-06-05

**Authors:** Marijana Kulić, Petra Bagavac, Marijo Bekić, Lovre Krstulović-Opara

**Affiliations:** 1 School of Medicine, 20000 Dubrovnik, Croatia; ravnateljica@dumed.hr; 2 Faculty of Electrical Engineering, Mechanical Engineering and Naval Architecture, University of Split, 21000 Split, Croatia; lovre.krstulovic-opara@fesb.hr; 3 Dubrovnik County Hospital, 20000 Dubrovnik, Croatia; marijob@bolnica-du.hr

**Keywords:** fracture, bone, ex vivo, biomechanics

## Abstract

This study investigates the mechanical behavior of femur bones under loading conditions, focusing on the transition from elastic to plastic deformation and eventual fracture. The force–displacement curves reveal distinct phases of deformation, with an initial linear relationship indicating elastic behavior, followed by deviation from linearity marking the onset of plastic deformation. Fracture occurs beyond a critical load, leading to a sharp drop in the force–displacement curve. The maximum fracture force varies among specimens and is influenced by bone geometry, size, cross-sectional area, and cortical thickness. Post-failure analysis highlights additional insights into fracture mechanics and bone material toughness. Reinforcing bones with screws enhances their strength, which is evident in the higher fracture forces observed in force–displacement diagrams. Fixation procedures following fractures further increase bone strength. Comparing specimens with and without strengthening underscores the effectiveness of reinforcement methods in improving bone mechanical properties. After analyzing the results, it is evident that femur bones with reinforcement can withstand greater loads, and they can also absorb higher impact energies while remaining in the elastic deformation range and without suffering permanent plastic damage. This study provides valuable insights into bone biomechanics and the efficacy of reinforcement techniques in enhancing bone strength and fracture resistance.

## 1. Introduction

The femur, the longest and most robust bone in the human body, plays a pivotal role in supporting posture and facilitating movement. It can withstand weights up to 30 times the mass of the human body, transferring forces from the head to the hip joint. The femur’s structure extends from the neck, passing through the greater and lesser trochanters, along its shaft to the medial and lateral condyles, ultimately connecting to the knee joint [1]. This bone is subjected to various types of loads, including compressive, tensile, bending, and torsional forces [2,3]. Factors influencing bone strength and quality encompass cortical geometry, cancellous tissue architecture, material microstructure, and tissue composition [4]. Despite its strength, the femur is susceptible to fractures, particularly in the neck or head region, often observed in athletes involved in high-impact activities or falls from height. Elderly individuals with osteoporosis are especially vulnerable to bending fractures in the shaft region. Given the substantial loads it sustains during daily activities, the femur can fracture under specific conditions.

Authors [5] investigated the merits of morphological and biomechanical of deer, pig and sheep bone as an animal model for the human femur. The morphological parameters measured include bone length, cortical and medullary diaphyseal diameters, cortical thickness, cortical cross-sectional area, and bone density along the diaphysis. Biomechanical tests included whole-bone four-point flexure tests to determine the bending stiffness (N/mm), Young’s modulus of bending (GPa), and ultimate strength in bending (MPa). On average, deer bone was found to be the least dissimilar from human femur. However, no single bone type consistently resembled the human femur. In this study, due to their relatively low cost and availability, fresh pig femurs were subjected to mechanical loading, offering insights relevant to both clinical and research settings.

Mechanical testing is clinically significant for assessing bone mechanical properties and structural integrity. The review article [6] shed light on how testing parameters impact the mechanical properties of mouse femur bones. It advocated for three-point bending over four-point bending and suggested a testing speed of 0.05 to 0.1 mm/s or even slower speeds. Although bone orientation during bending does not significantly sway results, the article advised maintaining consistency in orientation throughout testing for result comparability. Notably, differences exist between bones of female and male animals. Yet, after adjusting bone properties for body size, female mice demonstrate greater femoral maximum load, similar robustness, larger cortical area, and higher tissue mineral density (TMD) compared to male mice. Additionally, it suggests using bones from animals of similar age. The prevailing method for characterizing the biomechanical properties of appendicular long bones involves three-point bending testing of the midfemur in the anteroposterior (AP) direction. However, the elliptical cross-section of the femoral diaphysis is widest in the orthogonal mediolateral (ML) direction, suggesting that the femoral diaphysis should exhibit the highest resistance to bending along this axis. Authors [7] concluded that testing the mouse femoral midshaft in the ML direction is a precise and biologically valid method for determining the structural strength of this widely used skeletal site in experimental bone research. The study [8] involved analyzing the strength, stiffness, bone mineral density, and structure of the femur in both young and adult hares. Three-point bending test was performed to assess mechanical properties. The femora of young males exhibited higher elastic work compared to those of young females, while the femora of adult males exhibited higher elastic and breaking work than those of adult females. Similar research was conducted by the authors [9], concluding that there is significant correlation with bone stiffness and strength and age and sex. Female femurs exhibited significantly less strength, and also older bones tended to be significantly weaker for a given stiffness than younger bones. In this study, femur bones from young male pigs aged 5 to 6 months and weighing 100 to 120 kg were used.

An important concern in evaluating stresses in bone shafts stabilized by osteosynthesis metal plates arises after routine surgical procedures to repair severe bone fractures. Authors [10] induced this model in a femoral bone of an animal model, which was suitably stabilized with a dynamic compression plate (DCP) using bicortical screws. This system was submitted to bending to trigger damage in bone tissue in the vicinity of metal inserts. The developed procedure may be used to help surgeons to support decisions regarding bone repair using standard DCP. It is understood that bone regeneration following a fracture is significantly influenced by the stress conditions in the affected areas. Given that metallic implants such as plates and screws are commonly used to ensure fracture stabilization, assessing their impact on the induced stresses within the bone tissue becomes crucial. In this paper, the mechanical properties of healthy bones were compared with the properties of bones fixated with medical screws and bones fixated with one-third tubular plate. Ex vivo biomechanical bone testing using animal models is fundamental for comprehending bone health, injuries, and potential treatments.

## 2. Materials and Methods

In this experiment, the mechanical testing of fresh pig femur bone was performed. To avoid dehydration, the animal femurs were used fresh, within a maximum time of 2 days. During this study, in total, four tests were conducted, comprising two compression tests and two three-point bending tests. For each test, 3 fresh, healthy pig femur bones of approximately equal size were used, resulting in a total of 12 femur bones being used. Some femur bones were spray-painted and recorded during experiments with a high-speed camera for later digital image correlation (DIC) analysis. However, this is beyond the scope of this study and will not be further discussed here.

### 2.1. Pressure Test

**Figure 1:**
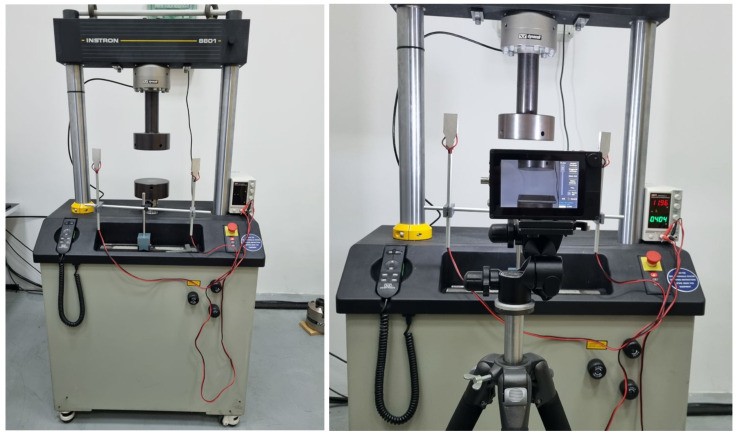
Universal hydraulic testing machine “INSTRON 8800”.

Pressure testing was carried out on the universal hydraulic testing machine INSTRON 8801 50 kN (Instron, Buckinghamshire, UK) (Figure 1). The lack of standardized tests can pose a challenge for researchers in assessing bone properties such as in the femur. Without a unified standard, researchers have to develop their own methods and approaches for measurement and analysis [11,12]. This can lead to variations in results and interpretations.

The femur bones were tested using a compression test to obtain the bone fracture force. Two sets of tests were conducted. In the first set, three bones were tested without reinforcement, while for the second set, three bones were reinforced with a medical titanium screw.

**Figure 2:**
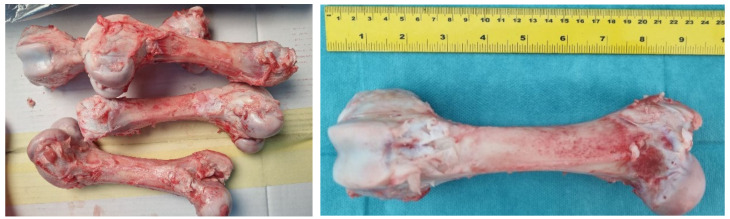
Cleaned femur (axis femoris).

Fresh pig femur bones were cleaned of meat and fat under controlled conditions. In Figure 2, the cleaned bones are shown.

**Figure 3:**
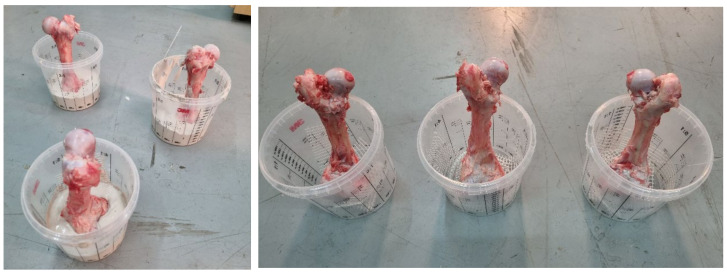
Lower part of femoral bone in plaster mold.

To ensure proper bone fixation and correct direction of external loading application in the hydraulic servo testing machine, the lower part of the bones was fixed in a plaster mold to help stabilize the trabecular free ends. In this position, they were supported by the lower head of the machine’s actuator, as shown in Figure 3.

The upper side of the bone was left without support, but to ensure that the axial force acted precisely at the correct location—the femur head—a metal washer was used during the experiment. A metal washer was chosen because stainless steel is a much stronger material than femur bone. This ensured that the measured deformation during the compression test came from the actual deformation of the femur bone and not from the deformation of the washer.

#### 2.1.1. Pressure Test of Femur Bone without Strengthening

**Figure 4:**
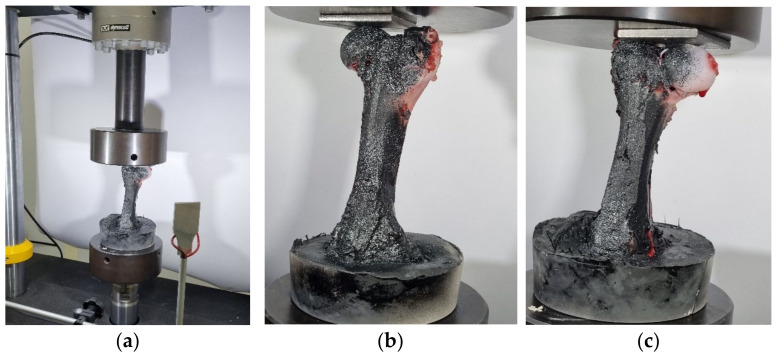
A compression test of a fresh pig femur bone: (**a**) start, (**b**) elastic deformation, and (**c**) plastic deformation.

Pressure tests were performed at a velocity of 0.05 mm/s, ensuring quasi-static loading. In Figure 4, a compression test of a fresh pig femur bone is shown at various time intervals. The femur bone fractured at the neck of the bone under the applied load, as shown in the figure. The fracture of the femur head, characterized by bleeding, is visible.

#### 2.1.2. Pressure Test of Femur Bone with Strengthening

**Figure 5:**
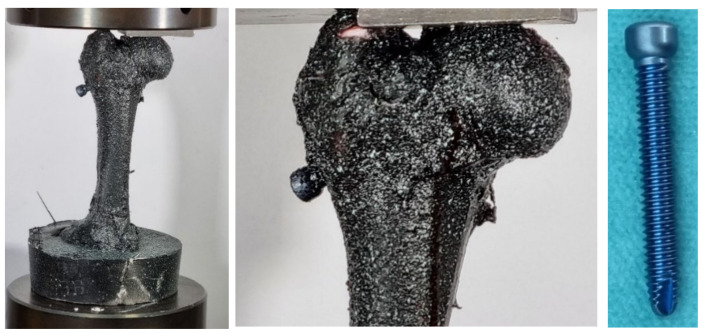
Strengthening titanium screws inserted in femur head.

Three pressure tests were performed at a velocity of 0.05 mm/s. Healthy femur bones were reinforced with medical titanium screws (86.620Txx Distal screw for Trochanter nail with a diameter of Ø6.28 mm and a length of 70 mm, manufacturer Instrumentaria d.d., Zagreb, Croatia). A titanium angled screw was placed through the femur neck, which gave additional reinforcement to the neck and head of the bone (a similar technique was applied when fixing the head and neck of the femur in orthopedics) (Figure 5).

### 2.2. Three-Point Bending Test

**Figure 6:**
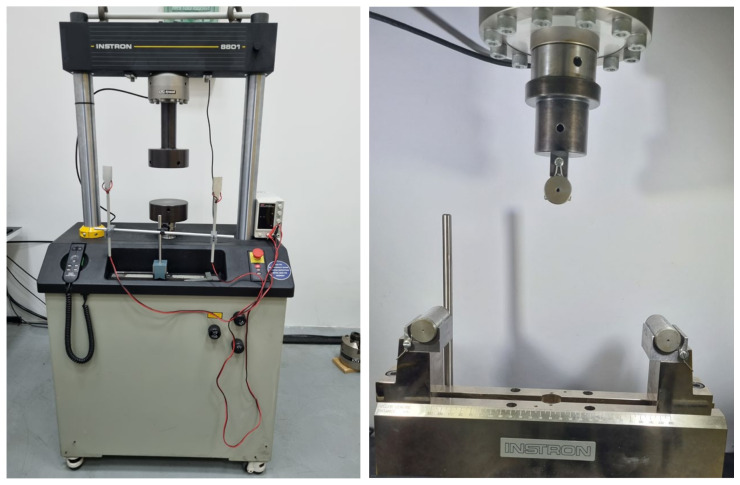
Mechanical claws for three-point bending test on universal hydraulic tearing machine “INSTRON 8800” with maximum force 50 kN.

Mechanical testing of femur bones using the three-point bending test is a common method employed to assess the mechanical properties and structural integrity of these bones [13,14]. The three-point bending test was carried out on the universal hydraulic testing machine INSTRON 8800 with a maximum force of 50 kN (Figure 6). In three-point bending test, parameters like stiffness (force over displacement) and strength (maximum load until failure) are directly measured and remain accurate, unaffected by beam theory limitations, because the cross-sectional geometry was not taken into account. However, these parameters can vary with support width. While a wider support width generally leads to reduced stiffness, it is crucial to note that results from one width may not accurately represent outcomes from another, requiring cautious interpretation and comparison of test results [15,16].

The femur is subjected to significant loads during daily activities and is prone to fracture under certain conditions. Understanding its mechanical behavior is crucial for various fields, including orthopedic surgery, biomechanical engineering, and forensic analysis. In the three-point bending test, a femur bone specimen was placed horizontally on two supports. A loading device applied a vertical force at the midpoint of the specimen, causing it to bend. This setup simulates the bending stresses that femur bones experience in real-world situations. The test typically involves applying incremental load until the bone fractures. During loading, measurements of force and displacement were recorded to generate a force–displacement curve, which provides insights into the mechanical behavior of the bone. During the experiment, the lower part of the bone was subjected to tensile stress, and in this region, the initiation of an initial crack was expected. When the cross-sectional area of the bone becomes too small to withstand the applied static force, bone fracture occurs. Mechanical testing of femur bones using the three-point bending test has important clinical implications. It helps in assessing bone quality, diagnosing conditions such as osteoporosis, evaluating the efficacy of medical treatments, and designing orthopedic implants and surgical procedures tailored to individual patients.

#### 2.2.1. Three-Point Bending Test of Femur Bone without Strengthening

**Figure 7:**
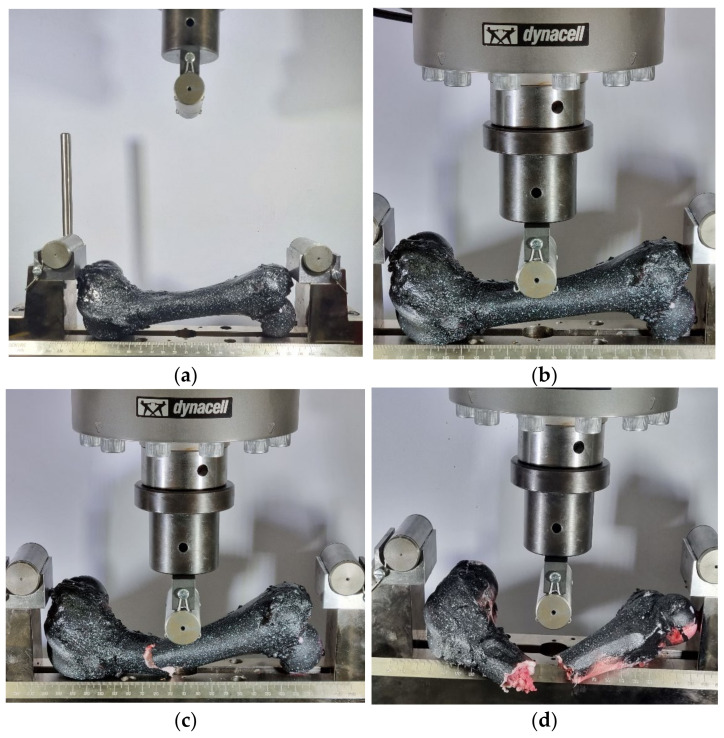
Three-point bending test of femur bone without strengthening: (**a**) before the start of the test, (**b**) first contact, (**c**) propagation of initial crack, and (**d**) fracture.

Three tests were performed at a velocity of 0.02 mm/s. In Figure 7, a three-point bending test is depicted at different time intervals. In Figure 7a, the bone is mechanically secured in the three-point bending fixture. To prevent bone slippage, a screw was attached to the head of the bone, ensuring its retention in the correct position throughout the test. Applying the indenter in three-point bending tests serves to focus the load at the center of the specimen, facilitating consistent and localized bending. While this may raise concerns about localized buckling, the test parameters and specimen fixture were chosen to minimize this effect and ensure accurate results. In Figure 7b, the first moment of contact between the middle roller and the axis of the bone is shown. Figure 7c depicts the fracture of the bone, displaying the progression from the initial crack to complete fracture. Figure 7d shows the complete fracture of the bone.

#### 2.2.2. Three-Point Bending Test of Femur Bone with Strengthening

**Figure 8:**
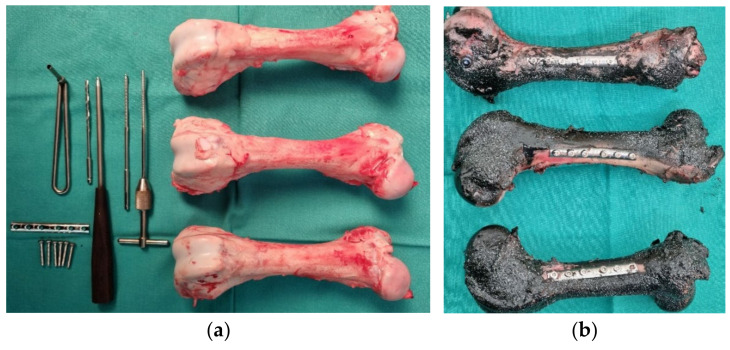
(**a**) Equipment for reinforcing femoral bone with a medical one-third tubular plate, and (**b**) reinforced femoral bones.

**Figure 9:**
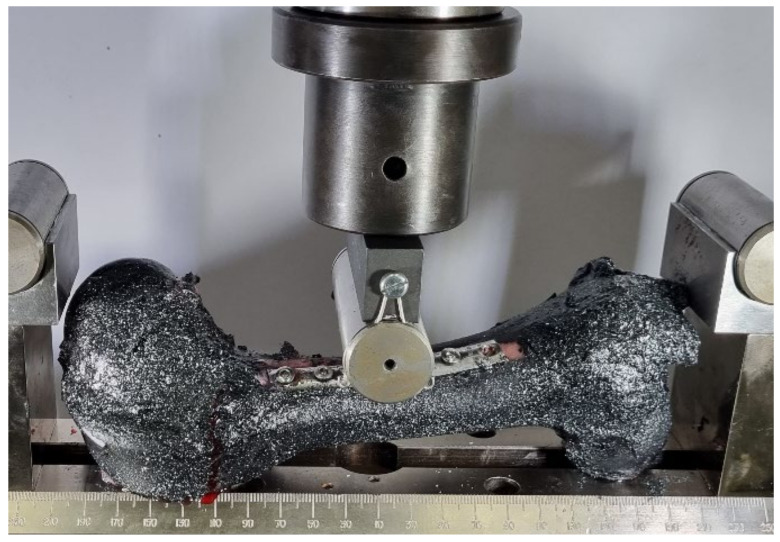
Application of concentrated vertical transversal load on reinforced bones.

In this experiment, the diaphysis of the femoral bone was reinforced with a medical one-third tubular plate (60.370-08 Plate for bones, length 97 mm, 1/3, manufacturer Instrumentaria d.d., Zagreb, Croatia) and six cortical screws (86.367-26 Cortical bone screw K-3.5, length 26 mm, manufacturer Instrumentaria d.d., Zagreb, Croatia). The equipment is shown in Figure 8a and three strengthened femur bones in Figure 8b. Three tests were conducted at a velocity of 0.02 mm/s. The application of concentrated load on reinforced bones is visible in Figure 9.

## 3. Results

### 3.1. Pressure Test of Femur Bone without Strengthening

**Figure 10:**
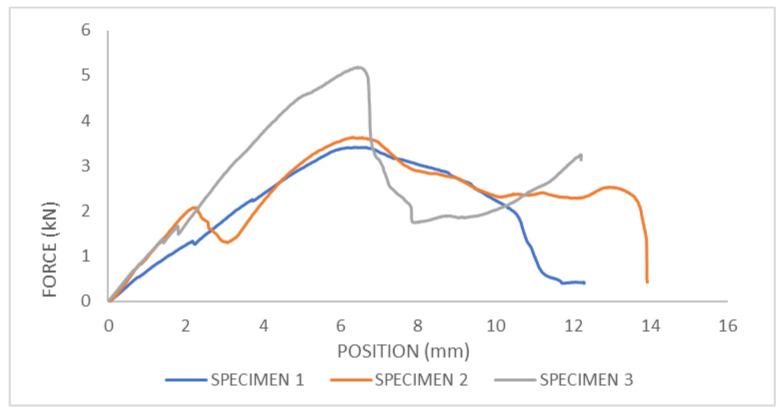
Force–displacement curve for pressure test of femur bone without strengthening.

At the beginning of the test, when the load was applied, the bone specimen deformed elastically, meaning it returned to its original shape upon removal of the load. In this phase, the force–displacement curve shows a linear relationship, indicating proportional loading and deformation. From the graph (Figure 10), it can be observed that during the application of the load, specimen 2 experienced a slip at one point (at the moment when the displacement is 2 mm).

As the load increases, the force–displacement curve may deviate from linearity, indicating the onset of plastic deformation. The point at which this deviation occurs is known as the yield point. Beyond this point, the bone begins to deform permanently. Eventually, the applied load reaches a critical value where the bone fails catastrophically, leading to fracture or complete failure. The force–displacement curve typically exhibits a sharp drop at this point, indicating failure of the bone specimen. From the force–displacement diagram, the maximum load at which bone fracture occurs can be determined. For specimen 1, the maximum load (fracture force) was 3.42 kN; for specimen 2, it was 3.64 kN; and for specimen 3, it was 5.19 kN. The maximum fracture force for all specimens occurred when the displacement of the upper jaw of the machine was 6 mm.

**Figure 11:**
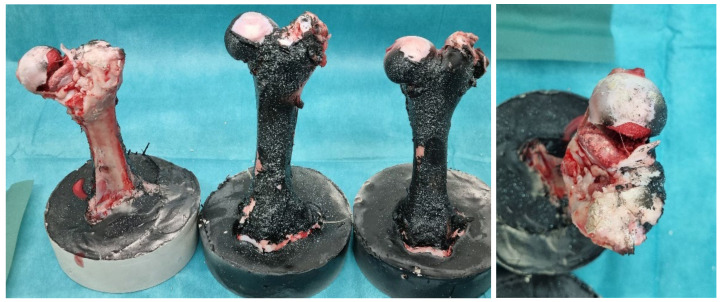
Femur bones without strengthening after the pressure test.

Fracture force depends on the geometry, size, cross-sectional area of the bone, and cortical thickness. Following failure, the force–displacement curve may continue with a slight increase in displacement due to the release of stored energy or secondary microfracture events. From the graph, it can be observed that after fracture, specimen 3 experienced a slip. Post-failure behavior can provide additional insights into the fracture mechanics and toughness of the bone material. In Figure 11, femur bones after pressure test and without strengthening are shown.

### 3.2. Pressure Test of Femur Bone with Strengthening

**Figure 12:**
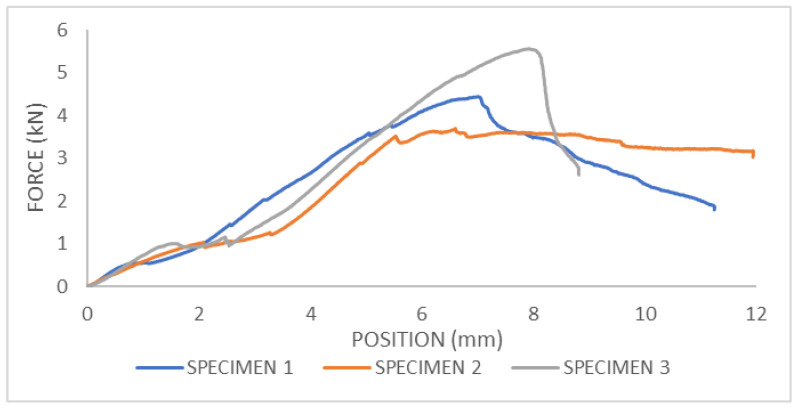
Force–displacement curve for pressure test of femur bone with strengthening.

**Figure 13:**
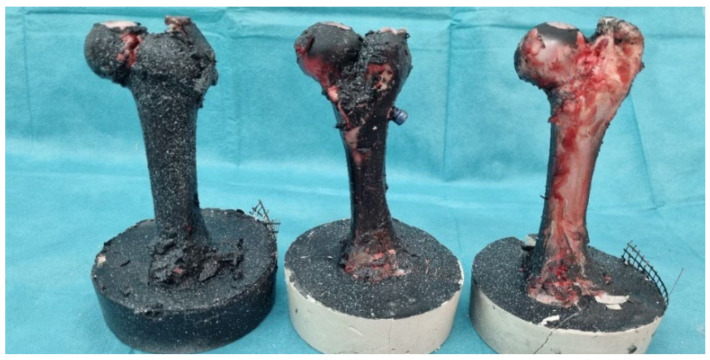
Femur bones with strengthening after pressure test.

During the study of reinforced bones, the aim was to determine the loading conditions of the entire bone in the neck area. This is because force is partially transferred from the bone’s head to the trochanter. Similarly, bone fixation is performed in cases of fractures, where the bone has fractured for any reason. Through this experiment, it was found that after inserting the screw, there was an increase in bone strength, as predicted. This was observed from the force–displacement diagram (Figure 12): The maximum fracture force for specimen 1 can be read as F = 4.45 kN; for specimen 2, F = 3.69 kN; and for specimen 3, F = 5.57 kN. In Figure 13, femur bones after the pressure test with strengthening are shown.

From the given results, it is evident that there was an increase in the strength of bones reinforced with a screw compared to bones that remained in their original state. Since the geometry of each tested bone is different, numerical results are quantitatively incomparable, but qualitatively, it is clear that the bones reinforced with a screw exhibit higher strength, thus demonstrating the benefit of fixation.

### 3.3. Three-Point Bending of Femur Bone without Strengthening

**Figure 14:**
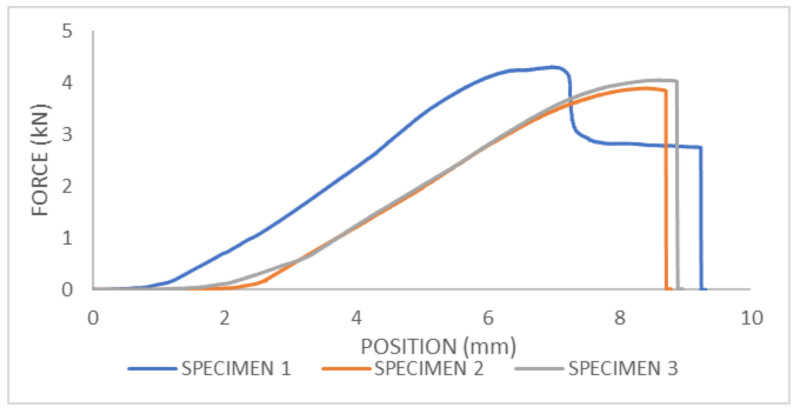
Force–displacement diagram for three-point bending test of femur bone without strengthening.

**Figure 15:**
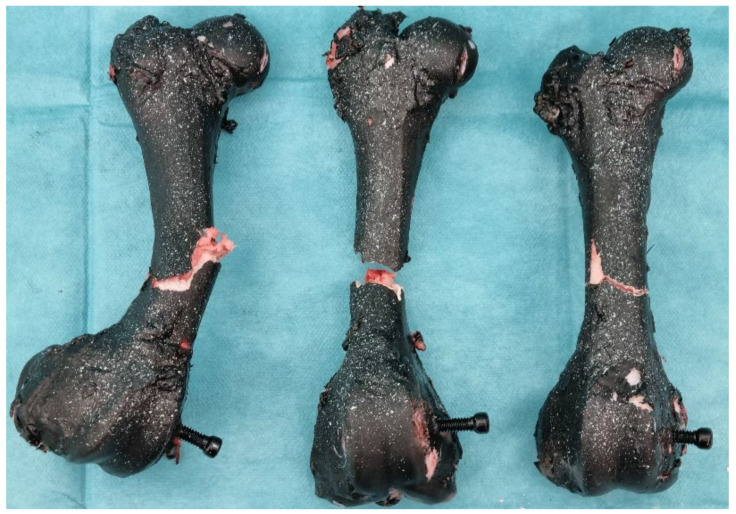
Femur bones without strengthening after the three-point bending test.

From the force–displacement diagram for femur bone without strengthening, the maximum fracture force in the three-point bending test can be obtained (Figure 14). For sample 1, the fracture force is F = 4.3 kN; for sample two, the fracture force is F = 3.89 kN; and for sample three, the fracture force is F = 4.05 kN. In the Figure 15, all three bones are depicted after the three-point bending experiment.

### 3.4. Three-Point Bending of Femur Bone with Strengthening

**Figure 16:**
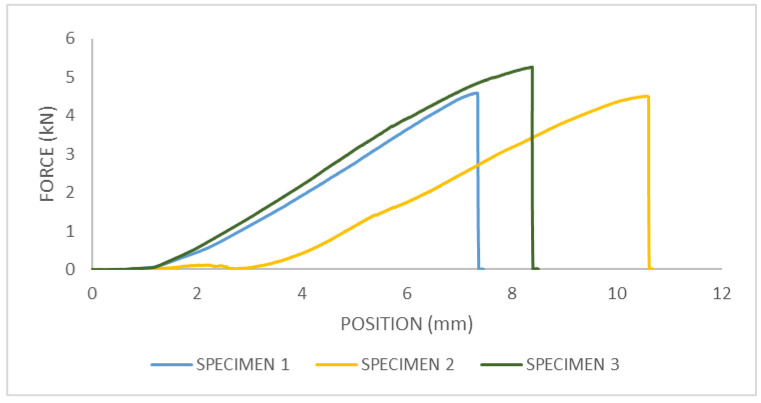
The force–displacement diagram for three-point bending test of femur bone with strengthening.

From the force–displacement diagram for femur bone with strengthening, the maximum fracture force in the three-point bending test can be obtained, Figure 16. For sample 1, the fracture force is F = 4.59 kN; for sample two, the fracture force is F = 4.51 kN; and for sample three, the fracture force is F = 5.26 kN.

**Figure 17:**
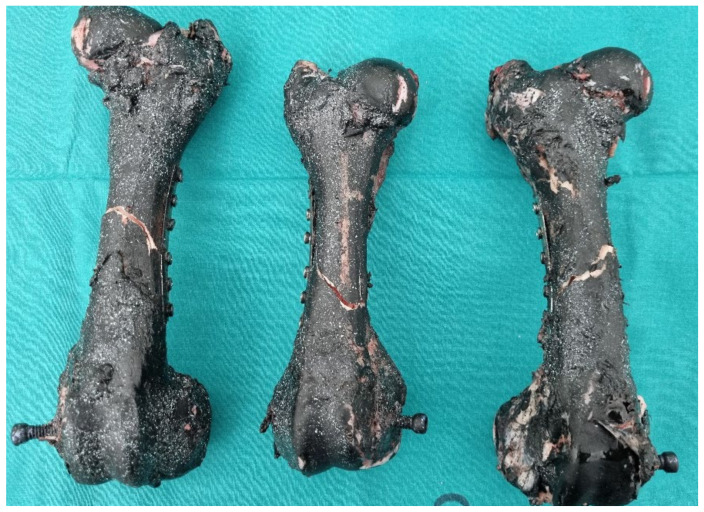
Femur bones with strengthening after three-point bending test.

After conducting the experiments, it was evident that a portion of the bone fractured due to concentrated loading (external vertical loading acting on the axis of the bone). Upon fracture, it was visible that the bone without reinforcement from the plate broke immediately below the point of concentrated loading, leading to the fracture of the bone itself. During the fracture of the reinforced bone (referring to the portion of the plate), it was noticeable that an increased fracture force was sustained, resulting in a minor fracture of the bone beneath the plate, as shown in the Figure 17. In the image, a clearly preserved portion of the bone at the fracture site is visible.

From the given results, it is evident that there is an increase in the strength of bones reinforced with medical one-third tubular plate compared to bones that remained in their original state. Since the geometry of each tested bone is different, numerical results are quantitatively incomparable, but qualitatively, it is clear that the bones reinforced with a screw exhibit higher strength, thus demonstrating the benefit of fixation with medical one-third tubular plate.

## 4. Discussion

The strength of bones is intricately linked to their material composition and structural integrity. Bones must possess a balance of stiffness to resist deformation and flexibility to absorb energy through deformation. Following failure, the force–displacement curve may exhibit a slight increase in displacement due to the release of stored energy or secondary microfracture events.

Upon conducting experiments, it becomes apparent that concentrated loading leads to the fracture of a portion of the bone. In cases without reinforcement, fractures occur immediately below the point of concentrated loading, whereas reinforced bones exhibit sustained fracture forces, resulting in minor fractures beneath the plate. These observations underscore the enhanced strength of bones reinforced with medical one-third tubular plates compared to their original state.

While numerical comparisons of the tested bones may be challenging due to differing geometries, qualitative analysis demonstrates the clear benefits of fixation with medical one-third tubular plates in increasing bone strength. Mechanical in vitro testing methods of long bones have been utilized for years to analyze mechanical behavior and strength, contributing to advancements in improving human skeletal integrity [17,18,19].

This experiment presents a series of tests conducted on femoral specimens obtained from postmortem pig donors, focusing on pressure and three-point bending tests with and without strengthening using fixation devices. It highlights specific behavioral patterns observed in the structural properties of bone-fixation constructs, considering the properties of both the fixation device and the bone itself.

Material properties, encompassing substances such as bone, stainless steel, and titanium, play crucial roles in determining construct behavior under load. Deformation of the construct under load is elastic, meaning it returns to its original shape upon load removal. However, overloading can lead to plastic deformation, characterized by permanent changes in shape [18,19,20].

Key properties of fixation constructs include the yield point, defining its safe functional load, stiffness in the elastic range, and fatigue resistance. Factors such as loading rate and age-related changes impact bone structure and mechanical properties, affecting load-bearing capacity, and will be part of future research work. External interventions such as proper nutrition, supplementation, training, and orthopedic fixation devices play vital roles in mitigating fracture risks associated with compromised bone strength [21,22,23].

In summary, a number of factors affect bone strength. Many external factors contribute to whole bone structure and strength; the proper use of these factors as preventive therapies, namely intake of essential nutrients such as calcium and vitamin D supplements, training and conditioning, and increasing bone strength using orthopedic fixation device, can minimize fracture risk in some affected individuals [23].

## 5. Conclusions

Through these tests, the maximum axial force, commonly referred to as the breaking point in mechanical engineering, that the femur can withstand was identified (Table 1). It is important to note that bone strength is not directly calculated in this context, as it heavily relies on the cross-sectional dimensions of the bone body (diaphysis). For meaningful comparisons of strength, bones with very similar dimensions were used.

The outcomes of these mechanical tests hold relevance in various fields, including biomechanics, orthopedics, and bone tissue engineering. The impact of load on the femur bone is contingent upon factors such as magnitude, direction, and rate of the load, with a proper understanding aiding in minimizing the risk of femur fractures in athletes and other individuals. This experimental setup mimicked the bending stress experienced by the femur bone in real-world scenarios. Incremental loads were applied until bone fracture occurred. The results, comprising force and displacement measurements, yielded a force–displacement curve, offering insights into the bone’s mechanical behavior. In the lower part of the bone, tensile stress is predominant, where the initiation of an initial crack is anticipated. Ultimately, when the cross-sectional area of the bone becomes insufficient to endure the applied static force, bone fracture ensues. Results show that femur bones with reinforcement can withstand greater loads, and they can also absorb higher impact energies while remaining in the elastic deformation range and without suffering permanent plastic damage.

## Figures and Tables

**Table 1:** Comparison of the strength and maximum displacement of the femur bone before and after reinforcement.

Test	Specimen	Maximum Force (kN)	Displacement at Maximum Force (mm)
Pressure test of femur bone without strengthening	Specimen 1.1	3.42	6.00
	Specimen 1.2	3.64	6.49
	Specimen 1.3	5.19	6.47
AVERAGE		4.08 ± 0.96	6.32 ± 0.28
Pressure test of femur bone with strengthening	Specimen 2.1	4.45	7.05
	Specimen 2.2	3.69	6.65
	Specimen 2.3	5.57	8.1
AVERAGE		4.57 ± 0.94	7.27 ± 0.75
Three-point bending of femur bone without strengthening	Specimen 3.1	4.3	6.65
	Specimen 3.2	3.89	8.1
	Specimen 3.3	4.05	8.87
AVERAGE		4.08 ± 0.21	7.87 ± 1.12
Three-point bending of femur bone with strengthening	Specimen 4.1	4.59	7.33
	Specimen 4.2	4.51	10.59
	Specimen 4.3	5.26	8.38
AVERAGE		4.79 ± 0.41	8.76 ± 1.66

## Data Availability

Data will be available upon request.
